# Oral Use of Phenytoin to Reduce Calcification in Bovine Pericardium and Porcine Aortic Leaflets Implants in Rats

**DOI:** 10.21470/1678-9741-2020-0252

**Published:** 2021

**Authors:** Mauro Barbosa Arruda Filho, Lamartine Daniel Aguiar, Silvio Romero de Barros Marques, Luiz Rafael Pereira Cavalcanti, Michel Pompeu B. O. Sá

**Affiliations:** 1 Division of Cardiovascular Surgery, Pronto-Socorro Cardiológico de Pernambuco (PROCAPE), Recife, Pernambuco, Brazil.; 2 Division of Cardiovascular Surgery, University of Pernambuco (UPE), Recife, Pernambuco, Brazil.; 3 Department of Surgery, Universidade Federal de Pernambuco (UFPE), Recife, Pernambuco, Brazil.; 4 Nucleus of Postgraduate and Research in Health Sciences, Faculdade de Ciências Médicas, Instituto de Ciências Biológicas, University of Pernambuco (UPE), Recife, Pernambuco, Brazil.

**Keywords:** Biocompatible Materials, Subcutaneous Tissue, Phenytoin, Calcium, Microscopy, Spectrophotometry, Atomic, Incidence, Calcinosis, Pericardium

## Abstract

**Introduction:**

This study aims to test the effect of phenytoin as an inhibitor of the process of dystrophic calcification in bovine pericardium and porcine leaflets implanted in the subcutaneous tissue of rats.

**Methods:**

Isolated segments of biomaterials were implanted subcutaneously in young rats. The study groups received 500 mg phenytoin per kilogram of diet per day. After 90 days, samples were collected and quantitative calcification assessment by optical microscopy, radiological studies with mammography, and atomic emission spectrometry were performed.

**Results:**

Inflammatory reaction was a frequent finding in all groups when analyzed by optical microscopy. The calcium level assessed by atomic absorption spectrophotometry was significantly lower in the study groups using phenytoin compared to the control groups (control bovine pericardium group X=0.254±0.280 µg/mg; study bovine pericardium group X=0.063±0.025 µg/mg; control porcine aortic leaflets group X=0.640±0.226 µg/mg; study porcine aortic leaflets group X=0.056±0.021 µg/mg; *P*<0.05). Radiologic studies revealed a statistically significant difference between the groups treated with and without phenytoin (not only regarding the bovine pericardium but also the porcine leaflets).

**Conclusion:**

The results obtained suggest that phenytoin reduces the calcification process of bovine pericardium segments and porcine aortic leaflets in subdermal implants in rats; also, the incidence of calcification in bovine pericardium grafts was similar to that of porcine aortic leaflets.

**Table:** 

Abbreviations, acronyms & symbols	
**GA**	**= Glutaraldehyde**
**Ig**	**= Immunoglobulin**
**L**	**= Control porcine aortic leaflets group**
**LF**	**= Study porcine aortic leaflets group**
**P**	**= Control bovine pericardium group**
**PF**	**= Study bovine pericardium group**

## INTRODUCTION

Calcification is a major cause of glutaraldehyde (GA)-fixed primary xenogeneic materials failure, mainly in children, leading to a high rate of reintervention^[1-3]^. The process and mechanism of calcification progression involving biological tissues in surgical practice is not yet fully understood. The process of calcification is related to the preservative used, due to its cytotoxic action, mechanical stress suffered by the heterograft, and host-related factors such as age, renal failure, and immune response^[4-6]^.

GA is undoubtedly the preservative of choice in the treatment of biomaterials. Nevertheless, the main commercially used preservatives are related to the promotion of dystrophic calcification by changes in collagen structure, amino acids extraction, presence of phospholipids that attract calcium ions, and the existence of cavities and gaps in the treated tissue produced by tissue fixation and cellular degradation^[7]^.

In an attempt to minimize these effects and increase the viability of xenogeneic grafts, several physical and chemical means have been associated after GA conservation, without consistent long-term results.

Phenytoin is an anticonvulsant drug synthetized in 1908. However, its non-sedative anticonvulsant properties were identified only in 1936^[8,9]^. Although its mechanism of action is not fully understood yet^[9]^, it is proven to promote changes in calcium metabolism and activity in dystrophic calcification by its antivitamin D action^[10]^.

This study aims to test the effectiveness of phenytoin as an inhibitor of the process of dystrophic calcification in porcine and bovine pericardium segments, preserved by GA and fixed by formaldehyde, implanted in the subcutaneous tissue of rats.

## METHODS

### Materials

All samples of bovine pericardium and porcine aortic leaflets used were commercial products from Braile Biomédica (São José do Rio Preto, São Paulo, Brazil). Fresh biomaterials were selected from healthy animals immediately after slaughter and rinsed several times with 0,9% sodium chloride.

The surrounding connective tissue was thoroughly removed. Each biomaterial was preserved by treatment with GA 0,65% (0.1 M HEPES buffer, pH 7.4, at room temperature, with two changes on the 2^nd^ and 7^th^ day) for 21 days. The biomaterial was thoroughly rinsed three times for 20 minutes and stored in alcohol solution (mixture of 1,2-octanediol, phenoxyethanol and 2,4-hexadienoic acid [1%] and ethanol [20%]).

### Animal Grouping

Eighty four-week-old male Wistar rats (100 g) received subcutaneous implants and were divided into two groups with forty rats each. Whereas the P group received a subcutaneous bovine pericardium implant, the L group received porcine leaflets implants. These two groups were subdivided again into study groups PF and LF (receiving phenytoin; each one with 20 rats) and control groups P and L (not receiving phenytoin; each one with 20 rats).

### Subcutaneous Implantation Technique

All procedures were in accordance with the rules of the *Guide for the Care and Use of Laboratory Animals* (Institute of Laboratory Animal Resources, National Academy of Sciences, Washington, D.C., 1996), respected the ethical principles on animal experimentation of the Brazilian College of Animal Experimentation (or COBEA), and were duly approved by the Ethical Committee on Animal Experimentation (or CEAA), Universidade Federal de Pernambuco.

**Fig. 1:**
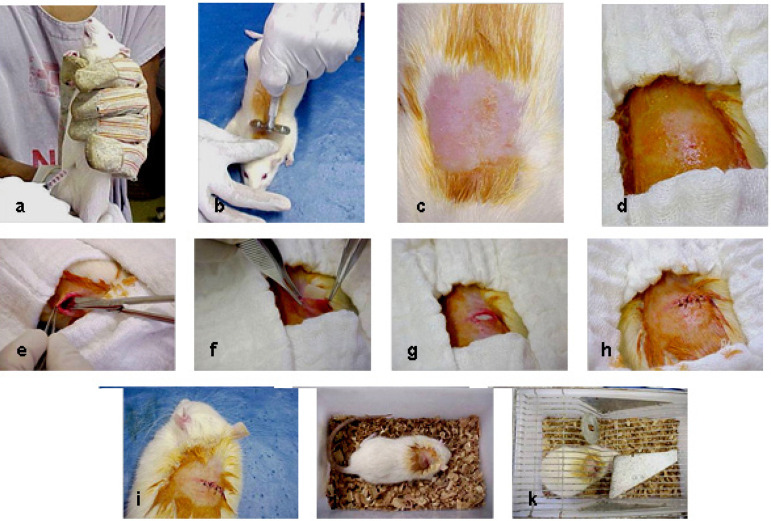
A - I) Step-by-step subcutaneous implantation technique; J - K) observation.

**Fig. 2:**
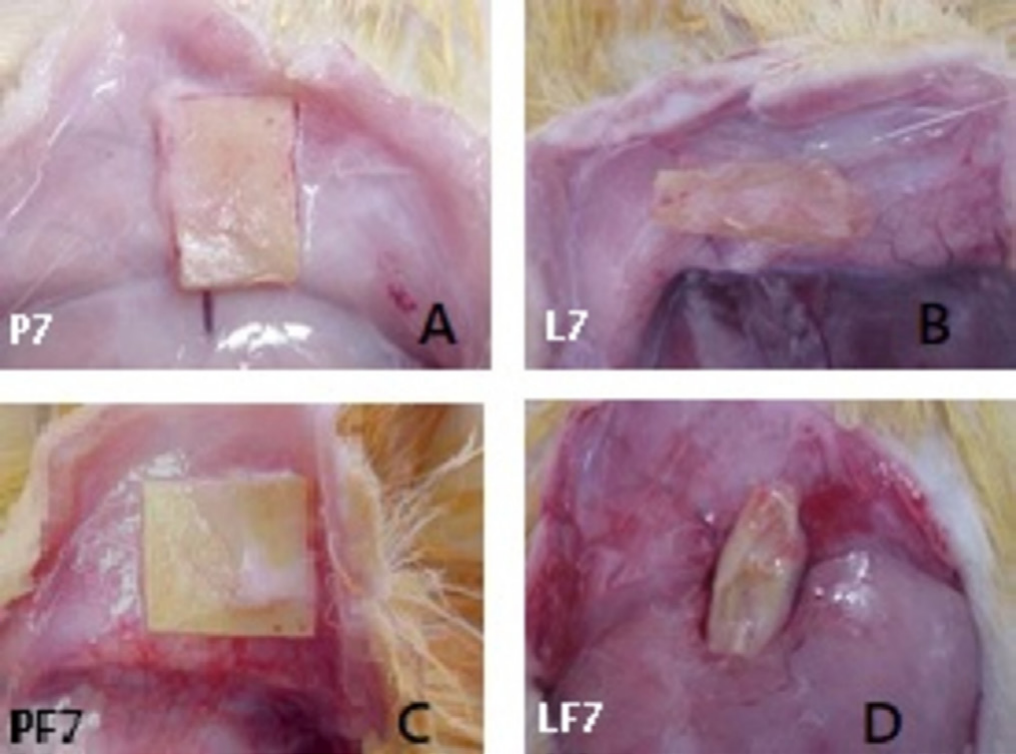
Samples explanted on postoperative day 90. A) Control bovine pericardium group (P); B) study bovine pericardium group (PF); C) control porcine aortic leaflets group (L); D) study porcine aortic leaflets group (LF).

The rats were anesthetized with 10% chloral hydrate at 0.4 mL dose per 100 g of body weight and the paravertebral region was shaved. A 1.0-cm long incision was made in the skin, dividing the subcutaneous tissue enough to allow the introduction and accommodation of 1 cm^2^ of biomaterial samples (Figure 1). After implantation, the animals were grouped in five per standard cage, where they were kept in daily observation, following the previously established feeding and medication program. Samples were explanted on postoperative day 90, divided into two parts, and then rinsed with 0.9% sodium chloride. One part was fixed in 10% neutral buffered formalin for histological studies, and another was dried at 60º C for calcium analysis (Figure 2).

### Phenytoin Dosage

The phenytoin dosage was calculated to be five times the recommended dose of the drug for pediatric patients. The daily dose was estimated to range from 10 to 12.5 mg, equivalent to a dose of 50 mg/kg of body weight (the average weight of rats at 90 days was 250 g). The drug was added to the diet of the PF and LF groups and the mice received the drug until the day of the tissue extraction - completing 90 days of dosage.

### Histological Study

**Fig. 3:**
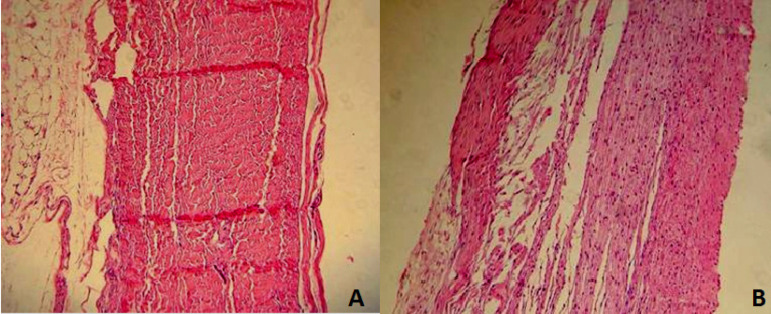
Normal histologic study. A) Bovine pericardium; B) porcine aortic leaflets.

For histological study, the parts were dehydrated in increasing concentrations of ethanol, diaphanized in xylene, and included in paraffin. Cuts were made four-micron thick and stained with Mayer’s hematoxylin-eosin and von Kossa’s method for minerals (Figure 3).

### Radiologic Analysis of Graft Calcification

After fixation with 10% formaldehyde solution, the grafts were submitted to analysis of calcification through mammography (a very sensitive radiological method). General Electric - Senographe DMR mammography device was used.

The level of calcification in radiological studies was divided into: (N) normal or equal to the fixed graft; (+) focal calcification, (++) segmental calcification, and (+++) intense calcification.

### Concentration of Calcium in the Grafts

After removal, the grafts were kept in 10% formaldehyde solution for 24 hours and then freeze-dried. After freeze-drying, the samples were immersed in a 6N HCl solution (at a rate of 3 mL of solution to 3 mg of freeze-dried tissue) and then hydrolyzed in a microwave system for model hydrolysis (MDS 2000 CEMCorp., Matthews, North Carolina, United States of America) for 45 minutes. After hydrolysis, the samples were diluted in a 5% 3N HCl solution. The calcium content of each sample was determined by atomic absorption spectrophotometry (AA-250Plus, PerkinElmer Inc., Norwalk, Connecticut, United States of America) and was expressed in micrograms per milligrams of dry tissue weight.

### Statistical Method

For statistical analysis of the qualitative variables, the Fisher-Freeman-Halton exact test was performed, and the data were transferred to InStat/MDR Software. For quantitative variables analysis, the Student's *t*-test was performed. The results were considered statistically significant when *P*<0.05.

## RESULTS

**Fig. 4:**
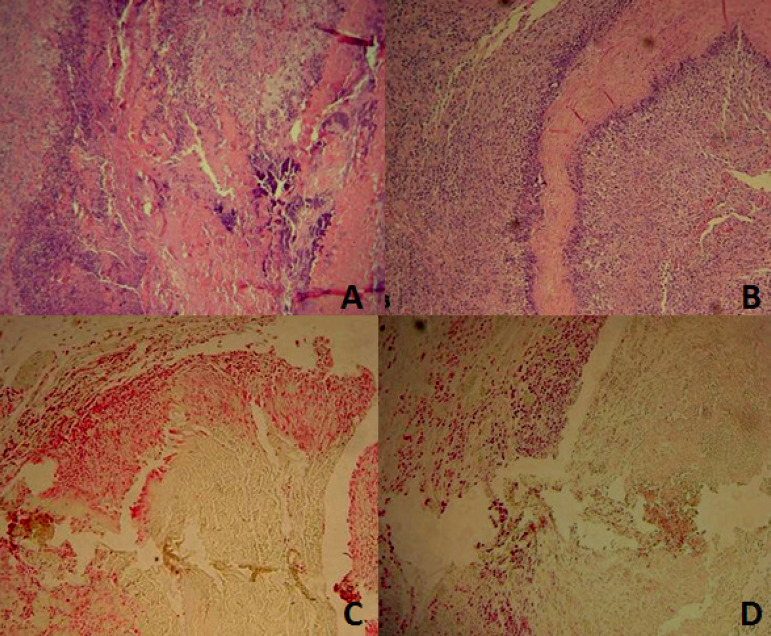
Light microscopy showing intense inflammatory reaction, with the presence of lymphocytes, histiocytes, and plasma cells in all groups studied. A) Control bovine pericardium group; B) study bovine pericardium group; C) control porcine aortic leaflets group; D) study porcine aortic leaflets group.

**Fig. 5:**
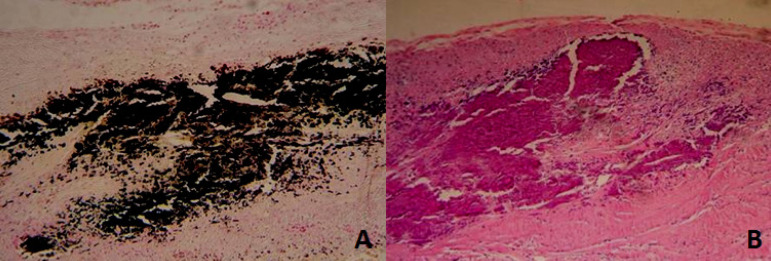
Histological calcification between groups. A) Study bovine pericardium; B) control bovine pericardium.

The evaluation by optical microscopy showed intense inflammatory reaction, with presence of lymphocytes, histiocytes, and plasma cells in all studied groups (Figure 4). There was a statistically significant difference regarding the decrease in calcification between the groups in the optical microscopy evaluation, despite a 5% reduction of calcification in PL and LF groups (Figure 5).

**Fig. 6:**
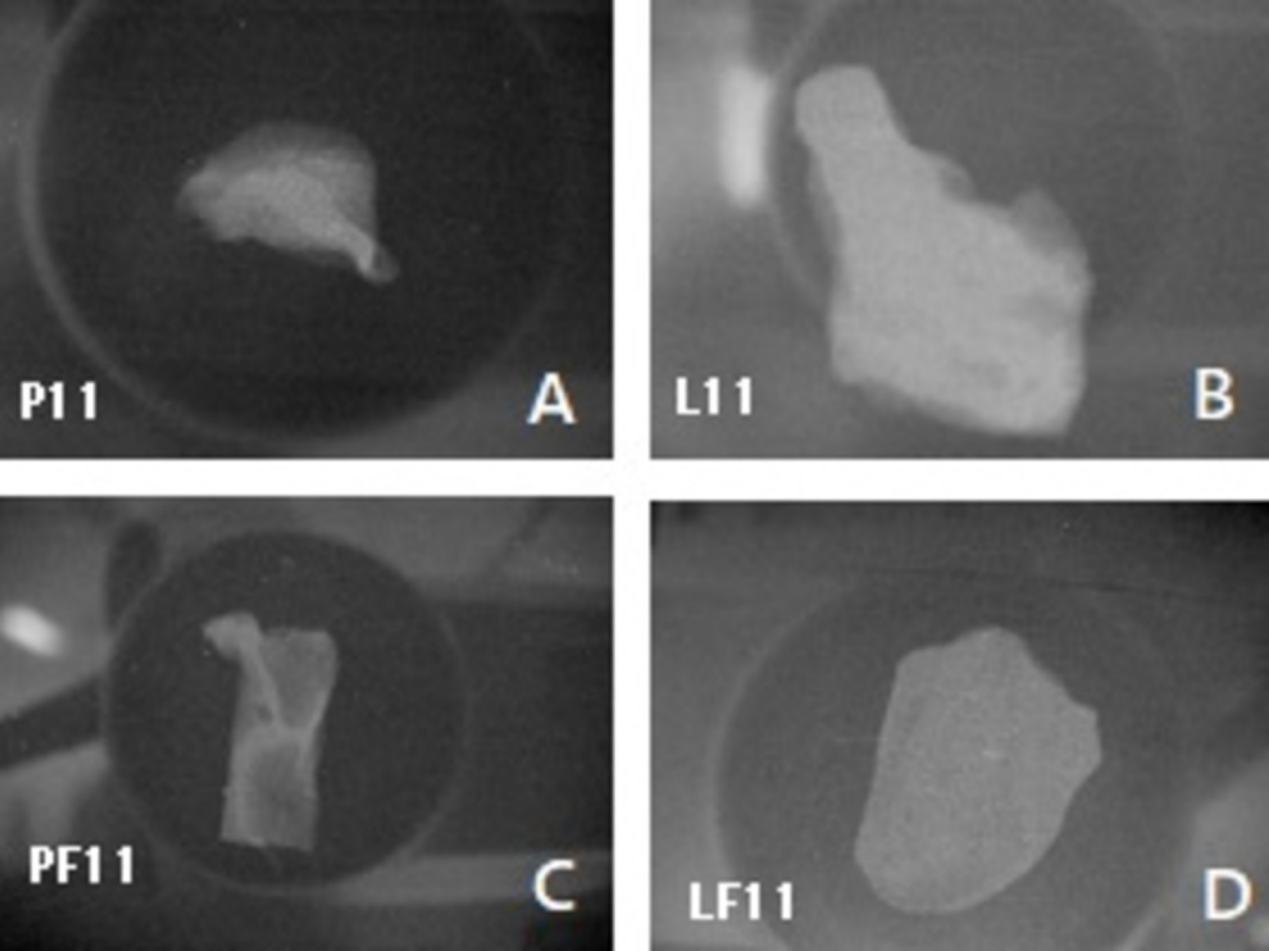
Mammography radiologic studies. A) Control bovine pericardium group (P); B) study bovine pericardium group (PF); C) control porcine aortic leaflets group (L); D) study porcine aortic leaflets group (LF).

**Fig. 7:**
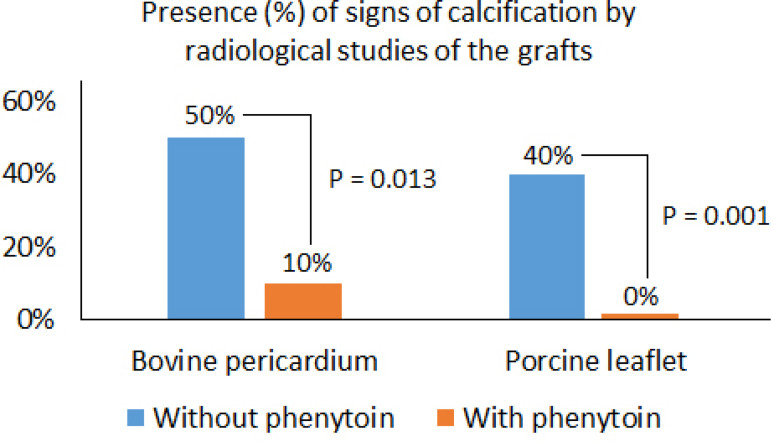
Presence of signs of calcification in radiologic studies of groups of bovine pericardium and porcine leaflets with and without phenytoin treatment.

Radiologic studies with mammography (Figure 6) revealed a statistically significant difference between the groups treated with and without phenytoin (not only regarding the bovine pericardium but also the porcine leaflets) (Figure 7).

**Fig. 8:**
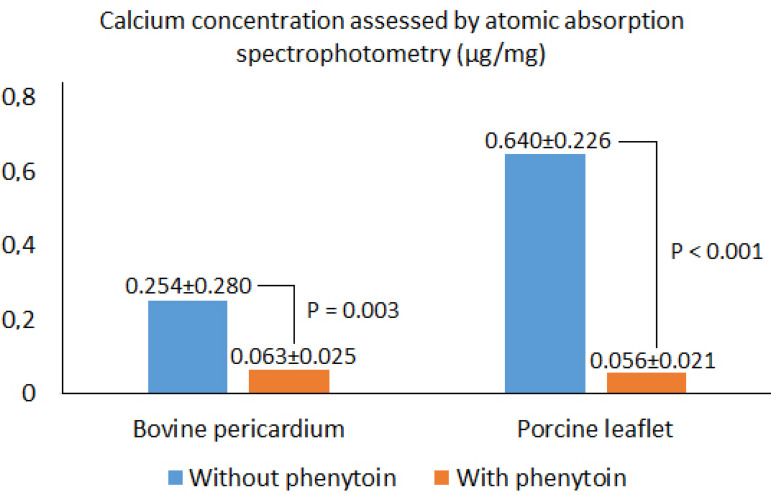
Calcium concentrations measured by atomic absorption spectrophotometry in groups of bovine pericardium and porcine leaflets with and without phenytoi

In this study, calcium concentrations measured by atomic absorption spectrophotometry were lower in PF and LF groups, which used phenytoin as an inhibitor of the calcification process up to 90 days after implantation. Among P and L groups, which did not received phenytoin, and PF and LF, receiving phenytoin as an anticalcifying treatment, there was no significant difference in calcium concentration (Figure 8).

## DISCUSSION

Dystrophic calcification of biomaterials and implanted bioprostheses can keep an intimate relationship with physiological calcification in bone formation^[11,12]^. The mineral deposit is basically the same in both processes. A deposit of hydroxyapatite crystals occurs initially in the matrix and, after exposure of these crystals to extracellular fluid, forms calcium crystals and later attracts the arrival of connective tissue, particularly collagen. These crystals become niche nurseries for the accumulation of more calcium crystals. Increased deposition of calcium crystals in active medullary formation depends on the availability of calcium and orthophosphate in extracellular fluid, and possibly the presence of collagen, which can be changed or delayed by the presence of natural mineralization inhibitors, such as proteoglycans, osteocalcin, phosphoproteins, and β-2-glycoprotein^[11,13]^.

Aortic porcine leaflets and bovine pericardium tissue are most commonly used in the manufacture of grafts for heart defects, fixed to intracardiac bioprostheses and valve replacements, being the ones chosen in this study for their routine use in current surgical procedures^[14,15]^.

The hypothesis that GA is one of the factors responsible for the process of calcification led researchers to develop process modifications and conservation studies, adding presumed mineralizing or inhibitory substances until replacement of fixatives^[11]^.

Vitamin D and the parathyroid hormones play a crucial role in regulating calcium metabolism, activating calcium-binding proteins, increasing their serum level, and acting on their intestinal absorption^[18]^. Low serum vitamin D levels reduce available calcium levels for bone formation and, therefore, reduce bone mass^[17]^. On the other hand, increased vitamin D serum levels can cause calcification of various other organs, but with minimal effect on the bones themselves^[18]^.

The increase in vitamin D metabolism can occur in young animals and in some human diseases, such as renal failure, infectious processes, and as an immune response of the host in the case of children and young people^[19]^. Age-dependent aspects of vitamin D metabolism may be linked to age-dependent aspects in heterografts and calcification of bioprostheses. Vitamin D is a stimulant for osteocalcin biosynthesis that is closely linked to the process of physiological and pathological calcification, and can interact to promote age-dependent calcification in GA-fixed tissue, through the action of osteocalcin^[20]^.

Phenytoin is a potent non-sedative anticonvulsant drug recommended by the American Epilepsy Society Guidelines to treat seizures unresponsive to benzodiazepines^[7,21]^. Its anticonvulsants properties are thought to stem from its activity in sodium and calcium channels^[7,8]^. Barnhart described that prolonged use of phenytoin may cause side effects such as osteomalacia, hypocalcemia, hyperphosphatemia, and increased parathyroid hormone concentration^[16]^. Phenytoin appears to exert an immunosuppressive effect on humans, reducing lymphocyte synthesis and proportional reduction in cells. It can cause a reduction in serum levels of immunoglobulin (Ig) G, IgM, IgA, IgE and decreased ability to demonstrate delayed hypersensitivity reactions, although these findings are dose- and time-dependent^[16]^.

Phenytoin’s antivitamin D effect occurs through increased induction of hepatic microsomal level of vitamin D catabolism and its metabolic effects and biological assets through a change in hepatic conversion of vitamin D (calciferol to 25-hydroxycalciferol)^[17]^. Liao et al.^[10]^ showed the effectiveness of phenytoin in inhibiting rat myocardial calcification and subcutaneously implanted bovine pericardium segments, when subjected to a special diet that enhances calcification, including myocardium.

The inhibitory action of calcification by phenytoin was to test phenytoin’s antivitamin D action in delaying the calcification process of xenomaterials implanted in subcutaneous tissue of rats. In our study, the phenytoin daily dose was set to be five times greater than human pediatric dosage. This addition was done on purpose, since the mouse metabolism is higher/faster than the human metabolism, it would enable a more effective assessment of phenytoin action, assuming that increasing the dose could have a greater action on calcium metabolism^[10]^.

The presence of fibrosis in the pathway, frequently found in histological studies (Figures 1 and 2), is related to the histological type of fixed xenograft used. The bovine pericardium is rich in type I collagen and the porcine aortic leaflets are rich in type III collagen, occurring more frequently in bovine pericardium than in porcine aortic leaflets^[11]^.

The evaluation by optical microscopy showed intense inflammatory reaction, with presence of lymphocytes, histiocytes, and plasma cells in all studied groups. The ability of GA to form covalent connections with proteins decreasing its antigenicity does not preclude the presence of intense inflammatory reaction in the literature reports, possibly because of its residual action^[20]^. These findings differ from those found by Liao et al.^[10]^, who suggested that the inflammatory reaction between the grafts and the GA-fixed host could be controlled with the use of phenytoin. There was a statistically significant difference regarding the decrease of calcification between the groups in the optical microscopy evaluation, despite a 5% reduction of calcification in PL and LF groups.

In this study, calcium concentrations measured by atomic absorption spectrophotometry were lower in PF and LF groups, which used phenytoin as an inhibitor of the calcification process up to 90 days after implantation. Some authors reported that the calcium concentration of GA-fixed grafts is progressive and related to the time of implant^[11,12,24]^, occurring more frequently at 45 days after implantation and being more dramatic at 90 days.

Among P and L groups, which did not received phenytoin, and PF and LF groups, receiving phenytoin as anticalcifying treatment, there was no significant difference in calcium concentration. These findings agree with those of Jorge-Herrero et al.^[24]^, suggesting that bovine pericardium tissues and porcine aortic leaflets are not prone to the calcification process. Both of which are used to make bioprostheses, with a greater tendency to use of bioprostheses made with bovine pericardium compared to those manufactured with porcine aortic leaflets^[25]^.

## CONCLUSION

The results found in this study (decreased incidence of macroscopic changes in consistency and staining, lower incidence of focal calcification and lower inflammatory reaction, lower incidence of calcification seen in radiological studies with mammography equipment, as well as by the lower calcium concentration, as measured by atomic absorption spectrophotometry, in PF and LF groups, which used phenytoin as an anticalcifying treatment) suggests that the antivitamin D action of phenytoin inhibited the process of dystrophic calcification.

**Table:** 

Authors' roles & responsibilities	
MBAF	Substantial contributions to the conception or design of the work; or the acquisition, analysis, or interpretation of data for the work; drafting the work or revising it critically for important intellectual content; agreement to be accountable for all aspects of the work in ensuring that questions related to the accuracy or integrity of any part of the work are appropriately investigated and resolved; final approval of the version to be published
LDA	Substantial contributions to the conception or design of the work; or the acquisition, analysis, or interpretation of data for the work; drafting the work or revising it critically for important intellectual content; agreement to be accountable for all aspects of the work in ensuring that questions related to the accuracy or integrity of any part of the work are appropriately investigated and resolved; final approval of the version to be published
SRBM	Substantial contributions to the conception or design of the work; or the acquisition, analysis, or interpretation of data for the work; drafting the work or revising it critically for important intellectual content; agreement to be accountable for all aspects of the work in ensuring that questions related to the accuracy or integrity of any part of the work are appropriately investigated and resolved; final approval of the version to be published
LRPC	Substantial contributions to the conception or design of the work; or the acquisition, analysis, or interpretation of data for the work; drafting the work or revising it critically for important intellectual content; agreement to be accountable for all aspects of the work in ensuring that questions related to the accuracy or integrity of any part of the work are appropriately investigated and resolved; final approval of the version to be published
MPBOS	Substantial contributions to the conception or design of the work; or the acquisition, analysis, or interpretation of data for the work; drafting the work or revising it critically for important intellectual content; agreement to be accountable for all aspects of the work in ensuring that questions related to the accuracy or integrity of any part of the work are appropriately investigated and resolved; final approval of the version to be published
